# Refining Ensembles of Predicted Gene Regulatory Networks Based on Characteristic Interaction Sets

**DOI:** 10.1371/journal.pone.0084596

**Published:** 2014-02-03

**Authors:** Lukas Windhager, Jonas Zierer, Robert Küffner

**Affiliations:** Institute for Informatics, Ludwig-Maximilians-Universität München, Munich, Germany; Tata Institute of Fundamental Research, India

## Abstract

Different ensemble voting approaches have been successfully applied for reverse-engineering of gene regulatory networks. They are based on the assumption that a good approximation of true network structure can be derived by considering the frequencies of individual interactions in a large number of predicted networks. Such approximations are typically superior in terms of prediction quality and robustness as compared to considering a single best scoring network only. Nevertheless, ensemble approaches only work well if the predicted gene regulatory networks are sufficiently similar to each other. If the topologies of predicted networks are considerably different, an ensemble of all networks obscures interesting individual characteristics. Instead, networks should be grouped according to local topological similarities and ensemble voting performed for each group separately. We argue that the presence of sets of co-occurring interactions is a suitable indicator for grouping predicted networks. A stepwise bottom-up procedure is proposed, where first mutual dependencies between pairs of interactions are derived from predicted networks. Pairs of co-occurring interactions are subsequently extended to derive characteristic interaction sets that distinguish groups of networks. Finally, ensemble voting is applied separately to the resulting topologically similar groups of networks to create distinct group-ensembles. Ensembles of topologically similar networks constitute distinct hypotheses about the reference network structure. Such group-ensembles are easier to interpret as their characteristic topology becomes clear and dependencies between interactions are known. The availability of distinct hypotheses facilitates the design of further experiments to distinguish between plausible network structures. The proposed procedure is a reasonable refinement step for non-deterministic reverse-engineering applications that produce a large number of candidate predictions for a gene regulatory network, e.g. due to probabilistic optimization or a cross-validation procedure.

## Introduction

Reverse-engineering of gene regulatory networks from gene expression measurements is applied to identify direct effector-target relations, i.e. to identify transcription factors binding to the promoter regions of genes to regulate gene expression (for reviews see [1]–[5]). Understanding the regulatory relations of an organism allows insights into its developmental processes, allows to predict expression changes as reaction to perturbations, and might eventually guide the development of diagnostic or therapeutic techniques. A major class of reverse-engineering algorithms are dynamical model based approaches which describe the actual effector-target relations by various mathematical frameworks (such as ODEs [6]–[8], Petri Nets [9]–[11], Boolean Nets [12]–[14]). The dynamical models can be created and optimized based on non-deterministic procedures involving iterative modifications of model structure and parameters (e.g. genetic algorithms, Monte Carlo methods, see [15], [16]). During optimization, models are repeatedly confronted with expression data from different perturbation scenarios (wild type, knockouts, overexpression, chemical treatment, etc.) and predicted gene expression levels are compared to experimental data to assess the validity of the models. The underlying basic assumption is that the ability to reproduce experimental observations correlates with the quality of a model, i.e. to be a good mathematical approximation of the biological system in question [17]. Putative effector-target relations can then be directly and easily derived from the model. The resulting network of interactions between pairs of genes constitutes a hypothesis about the (true) gene regulatory network.

### Ensemble Approaches in Reverse-Engineering

**Figure 1:**
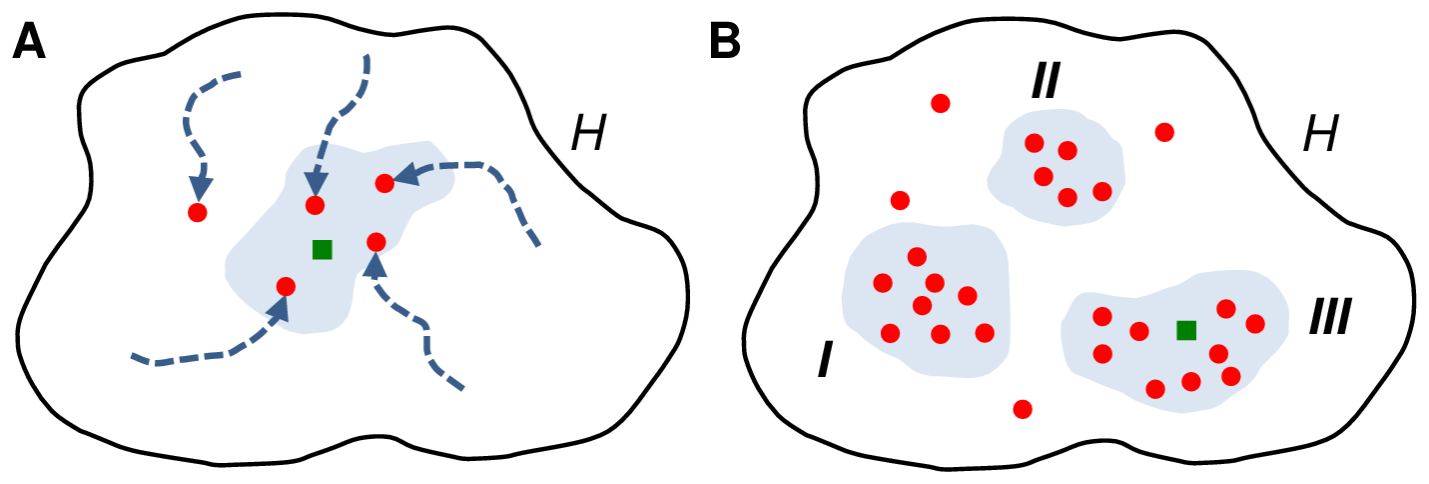
Reasons for ensemble averaging and its drawback. A) The hypotheses space *H* (black shape) contains all networks that can be represented by the applied mathematical framework. There might be no single optimum, as several different network structures might score equally well and thus are equally valid (blue area). Additionally, optimization procedures starting from different initial parameterization might get stuck at local optima and create suboptimal predictions (red dots). If the applied framework is adequate, the reference structure is included in *H* (green square) and could be predicted by the optimization procedure. Otherwise, predicted high scoring networks should be at least similar to the reference. Here, all high scoring predicted networks are very similar to each other and to the reference. In such a case, the frequency of an interaction in all networks is a reliable indicator for the confidence of an effector-target gene relation, thus applying ensemble voting is advisable. B) Depending on the reference structure, the applied mathematical framework, and the available experimental data, several groups of topologically different high scoring networks might be predicted by a probabilistic reverse-engineering algorithm (blue areas *I*, *II* and *III*). Combining all of these structurally strongly different networks by ensemble voting would obscure characteristics of individual groups of networks. We suggest the presence of sets of co-occurring interactions as a reasonable criterion for identification and delimitation of these groups.

Non-deterministic optimization is typically repeated several hundred or thousand times to collect high scoring networks, i.e networks that are able to reproduce the experimental data well. Most of these models are structurally different to each other, and none of them might be identical to the reference network. This is due to three fundamental reasons which might apply individually or jointly (adapted from [18]; figure 1A):

- representational: The applied mathematical framework is not suited to represent the true regulatory relations, e.g. due to simplifications. In such a case, also the best scoring predicted network might not be identical to the true gene regulatory network.
- statistical: Several different models reproduce experimental data equally well and, thus, lead to different, equally likely hypotheses. Even if a derived network equals the true regulatory network, the associated model can not be distinguished from others by its fit to the data.
- computational: The applied optimization algorithm might get stuck at high scoring local optima. Thus, resulting networks are sampled from suboptimal regions of the search space.

Nevertheless, it can be expected that interactions which are present in the reference network are at least enriched in high scoring networks. Thus, if a confidence for individual effector-target relations should be derived by reverse-engineering, it is more promising to consider the frequencies of interactions in all high scoring networks than to select a single prediction.

Several approaches have been proposed for this task and found to be superior in terms of precision, recall and robustness (see [19] and references therein). For example, voting schemes like majority voting, weighted voting, or signed voting can be applied to derive scores for each possible effector-target relation. These score (weight) is typically proportional to the frequency of an interaction in considered networks. In the case of weighted voting, scores might range from 1 (high confidence interaction) via intermediate scores (low confidence interaction) to 0 (high confidence non-interaction), where “high confidence” is synonymic to “observed in most/few considered networks”. High confidence interactions are apparently necessary to reproduce experimental data, as they are present in all high scoring networks. On the contrary, high confidence non-interactions might contradict experimental data, or their presence does not increase the fit of models but only its complexity and, thus, is disfavored. Low confidence interactions constitute variable sub-regions of networks, i.e. their functionality seems to be beneficial, but might as well be realized by alternative interactions. Therefore, these interactions are only present in a subset of high scoring networks while alternatives are present in others.

### Flaws of Ensemble Voting And How to Overcome Them

**Figure 2:**
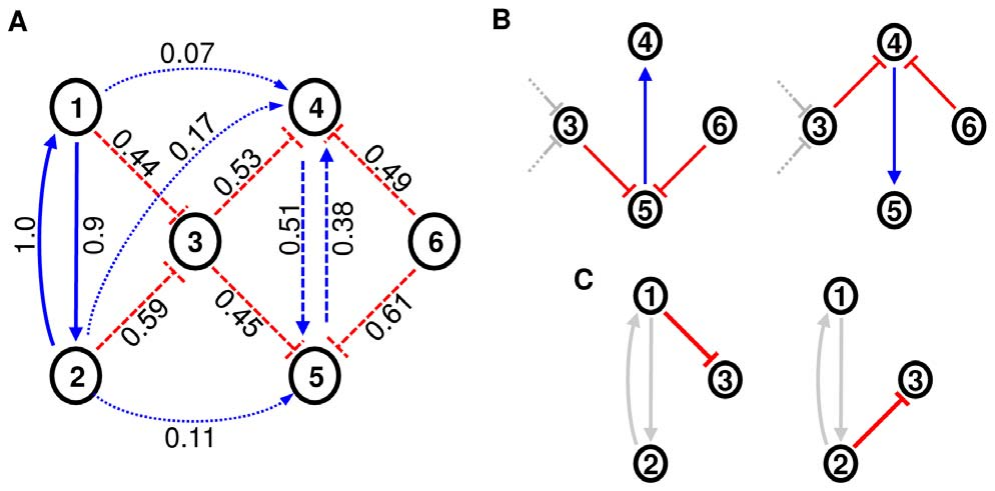
Illustration of an ensemble. An ensemble of several hundred predicted networks is created by calculating the frequencies of interactions. Reverse-engineering algorithms may produce suboptimal predictions, thus a certain amount of (random) variations in network topologies has to be expected. A) Ensemble average with annotated relative frequencies for activating (blue) and inhibiting (red) interactions. High confidence interactions (bold) are present in nearly all networks. High confidence non-interactions (dotted) are missing in most. Interactions present in subsets of networks have intermediate frequencies and are considered as low confidence interactions. B) Interactions connecting genes 3 to 6 constitute two characteristic sets (low confidence interactions). Either the left set of interactions or the right one is realized in predicted networks, but no mixture of sets or subsets. The co-occurrence of these interactions is not apparent in the ensemble. C) Interactions affecting gene 3 are mutually exclusive, but do not co-occur with other interactions. They can occur in combination with any of the characteristic sets and constitute an unspecific, highly variable sub-region of predicted networks.

Ensemble voting can be an adequate technique if considered networks are sufficiently similar to each other. However, if networks differ strongly in overall or local topology (figure 1B), then ensemble voting “lead[s] to a meaningless blur of alternative structures” [19]. In the following, we will discuss this flaw of ensemble voting in more detail and motivate our approach to overcome it. The discussion is illustrated by the small gene regulatory network of figure 2.

Assume that several hundred or thousand networks with different topologies have been created by a reverse-engineering algorithm due to statistical, representational, and computational reasons as described before. The interactions derived from all these networks can now be divided according to their frequencies: first, interactions that are present in nearly all networks.; second, interactions that are missing in nearly all networks; third, interactions that are present in subsets of networks only. When applying ensemble voting, the latter would constitute low confidence interactions (figure 2A).

Low confidence interactions can be further subdivided according to their mutual dependencies. First, interactions that co-occur with one or more other low confidence interactions; second, variable interactions without co-occurrence relations. Two interactions are co-occurring if the presence of one interaction is a reliable indicator for the presence of the other interaction and vice versa. In general, the presence of interactions is conditioned by the available experimental data, the chosen mathematical framework, and the applied reverse-engineering algorithm. Thus, the simultaneous presence of a pair of interactions seems to be necessary for a required functionality, i.e. only then a network can be high scoring. Obviously, some networks do not contain the set of co-occurring interactions, as otherwise these interactions would not be of low confidence. In these networks some competing set of low confidence interactions has to exhibit the otherwise missing functionality, and these interactions could be co-occurring as well. An example for competing sets of co-occurring interactions is given in figure 2B. Interactions that do not co-occur with others constitute highly variable sub-regions of predicted networks. They typically arise whenever multiple effector candidates for a single target exist that can not be distinguished using the available data, and thus can be freely exchanged. Interactions arising from multiple effector candidates might have a redundant functionality, so they increase the complexity of a model without increasing its fit to experimental data. Thus, such interactions are often mutually exclusive, although not necessarily (figure 2C).

If ensemble voting would be applied to all high scoring predicted networks, mutual dependencies would be obscured as associated interactions become indistinguishable from unspecific, highly variable interactions, and meaningful information would be lost. Thus, we propose that networks should be grouped according to the contained sets of co-occurring interactions (characteristic sets) and that ensemble voting should be performed separately on each group. Thereby, the interesting common characteristics can be preserved as co-occurring interactions would be enriched in the resulting group-ensembles. Thus, we utilize the presence or absence of defined sets of interactions as a local similarity measure.

Knowing the mutual dependencies between interactions and grouping networks accordingly has some clear-cut benefits. First and obvious, these dependencies between interactions become accessible for interpretation. Second, by grouping the typically hundreds or thousands of high scoring networks according to local similarities, the results of a reverse-engineering run become more interpretable. If ensemble voting is applied to each group, the fraction of low confidence interactions within each group-ensemble is decreased in favor of high confidence interactions. Third, guidance for the design of further experiments is provided. As either all interactions of a characteristic set or all interactions of the competing one are present, experimental validation of a single interaction should be sufficient to identify which characteristic set is actually realized in the biological system.

In the following, we present an approach for identifying mutually dependent interactions from a set of network predictions, combining co-occurring interactions to characteristic sets, and grouping networks according to the presence of these characteristic sets. We show that group-ensembles derived by an ensemble voting are superior to the ensemble of all networks in terms of interpretability, and that co-occurring interactions are especially suited for experimental verification.

## Methods

### A Characteristic Interaction Set Extraction Approach

The approach we present here consists of three subsequent steps: Calculation of interaction frequencies, derivation of scores for mutual dependencies, and finally grouping of networks. Due to the inherently high variability of networks caused by suboptimal predictions, a certain amount of *noise* has to be expected, i.e. redundant or missing interactions in any network. The input data is a set of high scoring networks predicted by a non-deterministic reverse-engineering algorithm. Each of these networks has the same number of nodes, representing genes, and a variable number of interactions, each representing a regulatory influence of an effector-gene to a target-gene. During the following explanation signs of interactions (activating or inhibiting) are omitted for simplicity. The according extension of the approach is straightforward and was applied for our evaluations.

#### Step 1: Interaction Frequencies

Each interaction 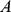 is classified according to its relative frequency 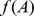 in all networks as

1: high confidence interaction if its relative frequency is above a cutoff,
2: high confidence non-interaction if its relative frequency is below a cutoff,
3: and low confidence interaction otherwise.

Only low confidence interactions are of interest for further processing, as stated in the introduction. For each pair of low confidence interactions 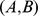 the relative frequency of its co-occurrence 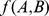 in all networks is calculated.

#### Step 2: Mutual Dependencies

For each pair of low confidence interactions a score for two mutual dependency relations is calculated as following:
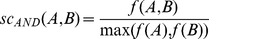


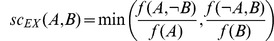
Where 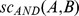 is a score for co-occurrence and 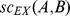 is a score for mutual exclusiveness of interactions 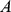 and 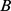. Hereby, 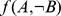 denotes the relative frequency of networks that contain interaction 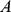 and miss interaction 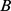. The relative frequencies as well as scores have to exceed respective cutoffs to consider a pair of interactions AND or EX related. Notice that scores are in the range 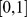 and if the score cutoff is 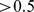, either 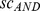 or 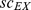 can exceed the cutoff but not both.

We define characteristic interaction sets as sets of AND related interactions. Individual AND related pairs of interactions constitute the initial characteristic sets. These two-element sets are then merged to characteristic sets of higher cardinality. Two characteristic interaction sets 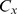 and 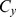 are merged if there is an AND relation between any 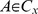 and 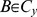. If there is an EX relation between any 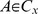 and 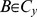, then the characteristic sets 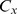 and 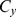 are considered competing, i.e. one of these characteristic sets can be present in a predicted network, but not both. Although it is possible that two characteristic sets have AND as well as EX relations between them, it was never observed during evaluations. Such rare conflicting cases should be resolved manually.

#### Step 3: Groups of Networks

All networks are then grouped according to the combination of characteristic sets they contain. E.g. if three characteristic sets 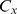, 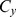 and 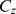 have been identified, where 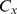 and 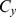 are competing, then there are five possible combinations allowed in predicted networks (only 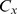, only 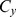, only 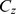, 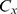 and 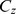, 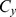 and 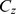). If no characteristic set is present a network is not considered for subsequent ensemble creation.

Ensemble voting can now be applied separately to each group of networks. Therefore, all interactions classified as low confident in step 1 that are part of the constituting characteristic sets are per construction enriched in the group-ensemble. Notice that each group-ensemble not only contains interactions from characteristic sets, but also all interactions previously classified as high confident, as well as other low confidence interactions.

## Results

To create networks for subsequent characteristic set extraction and validation, we applied a genetic algorithm (GA) to reverse-engineer dynamical models based on Petri Nets and Fuzzy Logic (PNFL, [20], [21]). This GA and PNFL were already successfully applied in the DREAM4 network reconstruction challenge [2]. In general, the performance of the applied reverse-engineering method is not crucial for the extraction procedure, as long as a sufficiently large number of models can be created, such that a few hundred fit the reference data well enough to be considered as (potentially) valid hypotheses.

**Figure 3:**
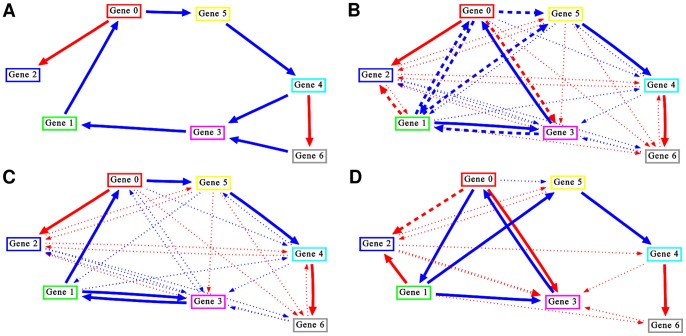
Example for extracted group-ensembles. Using simulated data from a random reference network (A), the applied reverse-engineering algorithm created a set of networks which were combined to an ensemble (B). Two group-ensembles (C,D) were derived using the described characteristic interaction set extraction approach. Both group-ensembles explain the simulated data very well (average RMSD 0.075 and 0.081) but effector-target relations differ strongly (AUPRC to reference 0.898 and 0.311). Blue lines: activating interactions. Red lines: inhibiting interactions.

Three hundred random gene regulatory networks were created for several different experimental settings reflecting different network sizes and an increasing amount of experimental data (table 1). Effector-target relations were considered to be either activating or inhibiting with equal probability and were assigned using a given in-degree distribution of 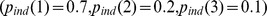, i.e. all genes had between one and three effectors. A wild-type time-series and the effects of a varying number of single and double knockout perturbations were simulated. For each reference, 1000 network predictions were created and the 20% with smallest root mean square deviation (RMSD) to the simulated reference data were used for characteristic set extraction. The following cutoffs were applied: interaction and non-interaction frequency cutoffs 0.8 and 0.1, joint frequency cutoff 0.1, AND and EX relation score cutoff 0.7. Using these cutoffs, characteristic interaction sets were found in the predicted networks of a varying fraction of references, depending on network size and available data (table 1). An actual example for reverse-engineered networks comprising a mixture of topologies is given in figure 3. Signed voting as defined in [19] was applied to create all ensembles. Contrary to the ensemble of all networks (figure 3B), co-occurring interactions are clearly visible in the group-ensembles (figure 3CD).

**Table 1:** Performance of reverse-engineering for varying network sizes and experimental settings.

A	B	C	D	E	F	G
5	3	0	1.3%	0.51/0.20	40%	75/39/7/0
7	4	0	0.6%	0.54/0.20	23%	51/16/2/0
10	5	0	0.0%	0.47/0.13	7%	19/03/0/0
15	8	0	0.0%	0.49/0.16	1%	03/00/0/0
5	5	0	6.0%	0.64/0.21	30%	60/23/5/3
7	7	0	1.0%	0.63/0.16	34%	74/22/5/1
10	10	0	0.0%	0.66/0.14	25%	59/12/4/0
15	15	0	0.0%	0.66/0.12	13%	34/05/0/0
5	5	3	12.0%	0.73/0.21	21%	47/16/1/0
7	7	4	3.7%	0.70/0.21	34%	68/30/4/1
10	10	5	0.3%	0.69/0.15	25%	58/14/3/0
15	15	8	0.0%	0.66/0.13	14%	36/06/0/0

For each of the twelve combinations of size and experimental setting, 300 random reference networks were created. For each reference, a wild-type time-series and a varying number of knockout perturbations were simulated. A) Number of genes in networks. B) Number of different random single knockout experiments simulated. C) Number of different random double knockout experiments simulated. D) Percentage of cases where a predicted network was identical to the reference. E) AUPRC of the ensemble of all networks (mean/standard deviation). F) Percentage of cases where characteristic interaction sets have been identified. G) Number of runs where 2/3/4/5 characteristic sets were identified. More than 5 sets were not observed.

**Figure 4:**
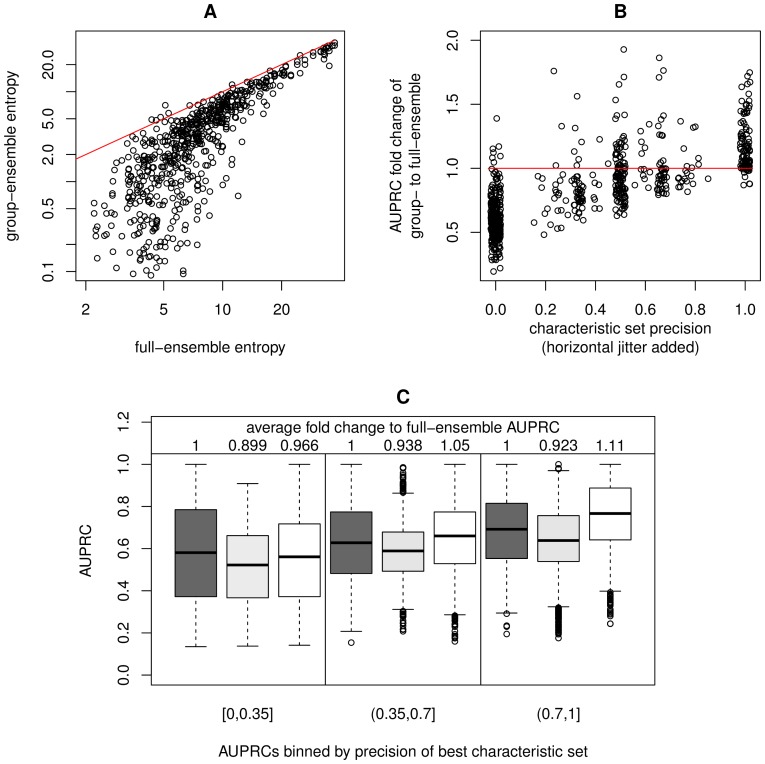
Entropy and AUPRC evaluation results. (A) The entropy of group-ensembles is on average decreased to 45% as compared to the entropy of the ensemble of all networks (full-ensemble). This is caused by the reduced fraction of low confidence interactions. (B) AUPRCs of group-ensembles are increased if their characteristic sets are present in the reference. Characteristic set precision ranges between 1 (all interactions are present in the reference) and 0 (no interaction is present in the reference). A small amount of horizontal jitter (<0.02) was added to the precision values for better visualization. The red lines indicate identity. (C) Rejecting alternative hypothesis by testing for the presence of characteristic set interactions (white boxplots) in general increases AUPRC, while testing for other low confidence interactions (gray boxplots) has a less pronounced or even negative effect. Thus, interactions that are predicted to be co-occurring with other interactions are preferred targets of further experimental verification. The full-ensemble AUPRC distributions are shown as dark-gray boxplots.

To show that grouping networks prior to ensemble creation decreases the fraction of low confidence interactions, the entropies of group-ensembles were calculated and compared to the entropy of the ensemble of all networks. An ensemble's entropy can be used as a measure of its overall confidence, i.e. ensembles with a large proportion of low confidence interactions (intermediate frequencies) have a higher total entropy compared to ensembles with many high confidence interactions (very high or low frequencies). Ensemble entropy is defined as 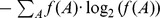, where 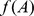 is the relative frequency of interaction 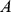. We found that group-ensemble entropies are on average reduced to 45% compared to the entropy of the ensemble of all networks (figure 4A).

Each created group of networks constitutes an alternative hypothesis about the true gene regulatory network, and one is interested to decide which of those is most similar to the reference. Typically, one would assume that the hypothesis which is superior in explaining experimental data is most similar to the reference. But only in 31.2% of the cases where two or more group-ensembles are found, the group-ensemble with highest similarity to the reference also has the smallest RMSD to the reference data. Hereby, the group-ensembles RMSD was calculated by averaging the RMSD of all contained networks. Additionally, the RMSD distribution of ensembles with highest similarity to the reference is not significantly different to the RMSD distribution of all other ensembles (Wilcoxon rank sum test, p-value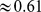). Thus, the score of a group-ensemble, i.e. the ability to reproduce the experimental data by the contained networks, is not suitable to decide for one of these hypotheses.

The similarity of an ensemble, i.e. a set of weighted interactions, to a reference network can be quantified by calculating the area under the precision-recall-curve (AUPRC). Hereby, interactions are sorted according to their frequency; precision and recall with respect to the reference are calculated for all frequency cutoffs; and finally the according area under the precision-recall-curve is derived. AUPRCs range between 1 (all reference interactions are top-ranked in the ensemble) and 0 (no reference interaction is present in the ensemble), thus they indicate the predictive quality of an ensemble. We checked whether some group-ensembles have an increased AUPRC as compared to the ensemble of all networks (figure 4B), thus are better predictions of the reference network than the ensemble of all networks. A positive correlation between AUPRCs of group-ensembles and the precision of characteristic sets was observed. Hereby, the precision of a characteristic set is the fraction of its interactions that could be found in the reference. This correlation can be expected, as characteristic interactions are per construction enriched in group-ensembles, and only if these characteristic interactions could also be found in the reference, then the AUPRC should increase due to this enrichment.

The networks which are most similar to the reference could be identified by further experimental evidence or additional prior knowledge concerning the presence of individual interactions. If the presence (or absence) of a certain interaction could be established, all hypotheses lacking (or containing) this interaction could be rejected. Interactions with intermediate frequencies are well suited targets for validation, as knowledge about their presence or absence would allow to reject a substantial proportion of networks by few experiments. Therefore, we simulated such experimental validations of individual low confidence interactions to test whether co-occurring interactions are especially suited for it. For each low confidence interaction, we checked whether it was actually present in the reference and accordingly rejected all networks lacking (or containing) the tested interaction. On average, the AUPRC of the ensemble of remaining networks increased only when testing for characteristic set interactions (figure 4C). Thus, it is beneficial for experimental design to distinguish between characteristic set interactions and other highly variable interactions beforehand.

## Discussion

When a new method for the reverse-engineering of networks is introduced it is often evaluated based on its precision to recover test networks with known underlying structures. It has been shown in the past that ensemble methods that generate a consensus by averaging over multiple predicted networks can improve this precision. However, crucial information from multiple predictions is lost in the consensus due to the involved averaging. In the present paper, we demonstrate that the alternative network predictions often form groups representing alternative hypotheses on the true network structure that cannot be distinguished given the currently available experimental measurements. Our proposed interaction set extraction approach first groups candidate networks and then performs ensemble voting separately on each network group. Instead of merely increasing the performance of inference, our approach thereby exploits ensembles of network predictions to extract and characterize such alternative hypotheses. This is an essential precondition to extend our understanding of a system's network structure, to facilitate future hypotheses tests and to enable the development of dedicated experiments that differentiate between otherwise equivalent hypotheses.

Our approach refines ensembles of networks as predicted by dynamic model based reverse-engineering algorithms. Thus, it depends on their prediction quality, proper sampling of models and the availability of a sufficiently large number of networks. These networks could be created by non-deterministic/probabilistic optimization, by separate optimizations based on different experimental data [22] or cross-validation, by incorporation of a varying amount of prior knowledge, or a combination of these. In our case, a non-deterministic genetic algorithm was used for network reconstruction.

The characteristic set extraction algorithm is intended to be simple, comprehensible, traceable and computationally fast. Pairs of co-occurring (AND related) and mutually exclusive (EX related) interactions are identified by calculating scores from their joint and individual frequencies. Notice that the typical number of observed effector-target relations is much smaller than the number of possible pairs, thus counting joint occurrences can be done very fast. Our definition of a score of AND relations was derived from the concept of confidence used in association rule learning [23]. The association rules 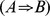 and 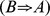 were combined by a *min* conjunction. The score of EX relations was defined analogously by combining 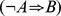 and 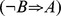 rules. Using the *min* conjunction is quite common in approximate reasoning (e.g. fuzzy logic [24]). Other conjunctions like *product* or *bounded product* are possible, but were not evaluated here. The interpretation of pairwise relations is straightforward. Both AND related interactions have to be present to achieve a biologically meaningful effect. EX related interactions might either represent redundancies, which are discouraged as sparseness of networks is typically demanded in reverse-engineering, or the presence of both interactions might contradict the reference data. Pairs of co-occurring interactions are then merged to characteristic sets of higher cardinality. This resembles a bottom-up procedure where large entities are constructed by joining of frequent smaller parts, and can be done very efficiently.

Cutoffs applied during characteristic set extraction are used to distinguish relevant observations (here, occurrence of certain interactions or characteristic interaction sets) from spurious ones, which might be artifacts of the applied reverse-engineering approach (noise). The specific choice of cutoffs is interdependent with the number and frequency of distinct characteristic sets hidden in the predictions. Additionally, the number of high-scoring networks with different topologies and, thus, the number of characteristic sets depends on the reference topology, available data, and the applied mathematical framework. Therefore, cutoffs have to be assessed case-specific, e.g. by starting with relatively stringent cutoffs, repeatedly reducing them and assessing extracted characteristic sets by number, size and biological meaning.

If one assumes that the only available information about the reference network are the time-series and knock-out measurements that were already used for reverse-engineering, we have shown that one can not easily decide which of the resulting groups of networks is more similar to the reference, as the average fit of simulated and experimental data does not allow such a distinction. Thus, the resulting groups of networks can not be reliably rated using the available information. One has to perform further experiments or otherwise gather additional information to rank these hypotheses or exclude some of them, e.g. by assessing the presence of individual interactions. Such additional information could be gathered using for example database searches, binding assays, perturbation measurements, text-mining, or expert knowledge. We have further shown that the design of additional experiments or the search for additional data can be guided based on the classification of interactions, i.e. interactions that are contained in characteristic sets are preferable targets for validation.

The presented procedure can be extended in various ways, e.g automatic adjustment of cutoffs, considering scores of predicted networks during ensemble voting to give high-scoring networks a higher influence, fuzzy assignment of interactions to characteristic sets and fuzzy grouping of networks to account for random variations, and using grouped networks as priors for repeated rounds of reverse-engineering and characteristic set extraction to explore different network hypotheses in more detail.

## Conclusions

If the topologies of predicted networks are strongly different, ensembles become blurry and obscure interesting characteristics of individual topologies. We propose to overcome this flaw by grouping networks according to the presence of sets of co-occurring interactions (characteristic sets). The resulting ensembles of grouped networks show an increased homogeneity and thus a smaller proportion of interactions with intermediate frequencies. This was quantified by a decrease of group-ensemble entropy to 45% with respect to the entropy of the ensemble of all networks. Group-ensembles constitute distinct, testable hypotheses about the system under consideration. They can be interpreted easily, as overall topology and dependencies between interactions become apparent. Thus, grouping networks according to characteristic topological features prior to ensemble voting is an advisable refinement step during reverse-engineering procedures that facilitates further experimental design and interpretation.
